# The Season, and Its Diseases

**Published:** 1854-07

**Authors:** 

**Affiliations:** Louisville


### Art. II.—THE SEASON, AND ITS DISEASES.

BY THE EDITOR.

We remarked upon the weather, in the early part of April, as being unseasonably cold. We have rarely experienced more uncomfortable days than some of the first of that month. On the 19th, according to the meteorological table of our friend, Lawrence Young, Esq., the temperature was as low as 24° Fahr. Since that time, however, there has been a great change, and we are now enduring the opposite extreme. Towards the latter end of June, after long continued rains, the weather became dry, and the thermometer rose to the height of our most intense summer heat. For ten days in succession the mean temperature was from 85° to 87°. On several days it was 96° and 97° in the afternoon, and was not below 80° at any time in the twenty-four hours.

The effect of this intense heat, supervening suddenly upon a rainy season, in a city not remarkable for its observance of hygienic laws, is interesting in an etiological point of view. One would say that here were present all the elements necessary to the production of disease. In Philadelphia or New York, half a century ago, the profession would have been on the lookout for yellow fever, under such a concurrence of circumstances. It was precisely such weather as would have excited apprehensions of cholera some years later. Added to the meteorological conditions, it is a noteworthy fact that cholera had made its appearance in many towns and villages not remote from Louisville early in the season; so that there were operating, at the same time, high temperature, moisture, decomposing animal and vegetable matter, and the poison of an epidemic disease at no great distance. Notwithstanding all, no serious form of disease has yet been developed in the city. Cholera infantum has been more prevalent than it has been seen before for a number of years. Cholera morbus and diarrhea have been of frequent occurrence, and there have been a good many cases of dysentery. But nothing bearing any resemblance to an epidemic has prevailed; the city cannot even be said to have been sickly. Cases of cholera have been brought to our wharves by steamboats, every few days, since the opening of summer, and a few persons have died in different parts of the city with symptoms strongly resembling those of cholera. Still the disease has not spread, and up to this time has manifested no tendency to become epidemic.

How long this favorable state of the public health will continue under circumstances apparently so unpropitious, cannot, of course, be foreseen; but we confess it has gone far to confirm a conviction which has been for a good while gaining strength with us, that the weather does not materially affect the progress of cholera. Not that cholera prevails in all seasons alike; the whole history of the pestilence disproves this. It is eminently a disorder of summer. Cold weather almost always arrests it. But what we mean to say is this, that during the warm season it appears to be quite independent of the weather. We remember well that it broke out in this city, in the summer of 1851, when the clouds were pouring down torrents of rain and a sultry state of the atmosphere prevailed, and that it subsided as suddenly as it had come on, in less than a week, while the rains were as copious as ever, and the thermometer indicated high summer heat. It made us doubt whether cholera was dependent upon the weather. We had begun to doubt it before. We had seen Lexington ravaged by the pestilence in 1833, while Versailles, a town only twelve miles away, escaped it; and, in 1835, we had seen Versailles desolated by the malady, while Lexington enjoyed the most perfect health. How are these things if cholera is a disease of the weather? In the year succeeding the one when the epidemic was so fatal, the summer opened upon Lexington warm and wet, and the public mind was alarmed at the prospect of a second invasion; but though the weather was pronounced "cholera weather," no cholera appeared. How many wet, warm seasons have returned since then to that beautiful city we know not, but it is well known that it was never revisited by cholera again until the plague had again become epidemic in the country, in 1849.

Dr. CARROLL, of Cincinnati, in an able paper on Cholera, in the *Western Lancet*, gives the following table of the weather, which will be seen to favor the view which we have expressed:—

| | May. | June. | July. | August. |
|---|---|---|---|---|
| Mean temperature in 1849 | 63° 9' | 73° 9' | 73° 7' | 73° 5' |
| same 1850 | | 73° 26' | 81° 65' | 78° 26' |
| same in last 15 years. | 63° 6' | 70° 9' | 75° 1' | 73° 1' |
| | inches | inches | inches | inches |
| Rain in 1849, | 3.61 | 4.90 | 8.90 | 4.41 |
| do 1850, | | 5.00 | 6.30 | 7.20 |
| do mean amount in last 15 years. | 5.02 | 5.43 | 4.58 | 4.47 |

Thus, in 1849, after a June more dry than usual and with a temperature below the average, cholera was prevailing with the greatest fatality during the first days of July. Indeed, Dr. CARROLL believes that it was as fatal by the 15th of June as at any future time, and on this point he remarks:—

"Comparing July '49 with July '50, the mortality of the latter year should have been much greater than in the preceding one—that is, if extreme heat could decide the matter; for we find the mean temperature more than 8° higher than that of '49. Yet the fatality of the disease was not so great, or rather, the number of cases was much less. It will be seen hereafter, however, that the cases which did occur in '50, particularly those in a collapsed state, were less under the control of medicine than those of the former year. There was but little difference in the June of the two years, either in the quantity of rain or the temperature, yet the mortality of June '49 was appalling, whilst in '50 we were almost exempt from the malady until the approach of July. But a comparison of August in the two years is more conclusive than either of the others. In the latter year, the temperature of this month was five degrees higher, and the amount of rain nearly two thirds greater than in '49; yet we find the cholera almost extinct in '50, whilst in the same month of the preceding year, it still continued to destroy, though much mitigated in violence.

It would be an easy matter to multiply instances of this sort, but it is not our intention to go fully into the argument at this time. The more the subject is examined the stronger, we think, the proof will appear, that cholera depends upon some poison which heat and moisture may intensify but will not always develope, and that while it is affected by the weather it operates at all seasons and in all varieties of weather.

So far, this season, cholera has exhibited more of the character of a sporadic disease than of an epidemic. The places where it has appeared are very numerous, but from none has it spread extensively, nor has its fatality, at any point, been comparable to that which marked the progress of the epidemic in former years. In a few small towns the mortality has been considerable for a few days, but the pestilence has been of brief duration. Whether such will continue to be the course of the plague through the season cannot be predicted with certainty, but there are certainly a good many reasons for hoping that it will.

Physicians with whom we have conversed are of the belief that those cases of cholera, or of cholera morbus approaching in severity to the genuine disease, with which they have met, have been more tractable than they found similar cases when the plague was epidemic, and such has also been our experience. We have seen cases of profuse vomiting and purging, attended by violent cramps, and a corrugated skin, relieved promptly by the free use of opiates, with chloroform by inhalation and given internally. Chloroform appears to us to be an agent of great value where cramps, as is often the case, constitute the most distressing symptom.

In cholera infantum some practitioners have prescribed quinine with very satisfactory results.

Dysentery has not prevailed so extensively as it did two summers ago and has not been fatal. The anodyne practice, we believe, has been universally pursued. Some form of opium has been given to the extent of allaying the tenesmus, restraining the alvine discharges, and making the patient, as far as possible, comfortable.

In all these diseases, cold water and ice have been allowed liberally, and have been found most grateful and useful remedies.

The above was written four days ago. Since that time a most grateful change has occurred in the weather. Our mornings for several days past have had a temperature of 70°, and cool breezes from the North-east have assuaged the intense heat of the day. The weather is now as fine as could be desired—the health of the city excellent.

Cholera continues to be a topic of some interest—not, however, an engrossing topic—in most of the cities and towns of our country. From New Orleans to Boston it is reported as having its daily or weekly victims in nearly all the principal cities. Among the immigrants in St. Louis the fatality from the disease was serious at the last dates. In New York it was slightly on the increase, but nothing has yet occurred to excite apprehensions that the pestilence is about to assume an epidemic character in that city.

The fact that cholera has appeared in so many places, remote from each other, many of them in the interior and comparatively sequestered, far away from the avenues of travel and commerce, shows that it depends upon some general zymotic influence, and not at all upon the importation of a contagion. Immigrants are the greatest sufferers from the disease, but although they have been coming to New York and New Orleans with the plague among them for the last two summers, it has not spread and become epidemic.

We think we may now cease to look upon this disease as an erratic visitant from foreign parts, and begin to regard it as one native to the soil, which is engendered by causes not yet well understood but local in their character.

That it has been, so far this season, much less malignant than during former visitations, seems to be a fact universally conceded, and it is pleasant to observe that the advent of the disease no longer creates those panics which, in years past, everywhere marked its irruption.

During the intensely hot days of which we have spoken, a number of cases of *sunstroke* occurred in this city, some of which terminated fatally. Cases of this affection are reported to have been frequent in St. Louis, New York, and New Orleans, about the same time, the weather in those cities having been even more intemperate than here.

Louisville, July 14, 1854.

